# S-Nitrosylated hemoglobin predicts organ yield in neurologically-deceased human donors

**DOI:** 10.1038/s41598-022-09933-z

**Published:** 2022-04-22

**Authors:** Ryan Nazemian, Maroun Matta, Amer Aldamouk, Lin Zhu, Mohamed Awad, Megan Pophal, Nicole R. Palmer, Tonya Armes, Alfred Hausladen, Jonathan S. Stamler, James D. Reynolds

**Affiliations:** 1 Institute for Transformative Molecular Medicine, School of Medicine Case Western Reserve University, Cleveland, OH USA; 2 Department of Anesthesiology and Perioperative Medicine, School of Medicine Case Western Reserve University, Cleveland, OH USA; 3 Department of Pulmonology and Sleep Medicine, School of Medicine Case Western Reserve University, Cleveland, OH USA; 4 Department of Medicine, School of Medicine Case Western Reserve University, Cleveland, OH USA; 5 Harrington Discovery Institute, University Hospitals-Cleveland Medical Center, 4-128 Wolstein Research Building, 2103 Cornell Road, Cleveland, OH 44106 USA

**Keywords:** Biomarkers, Outcomes research, Translational research

## Abstract

Current human donor care protocols following death by neurologic criteria (DNC) can stabilize macro-hemodynamic parameters but have minimal ability to preserve systemic blood flow and microvascular oxygen delivery. S-nitrosylated hemoglobin (SNO-Hb) within red blood cells (RBCs) is the main regulator of tissue oxygenation (StO_2_). Based on various pre-clinical studies, we hypothesized that brain death (BD) would decrease post-mortem SNO-Hb levels to negatively-impact StO_2_ and reduce organ yields. We tracked SNO-Hb and tissue oxygen in 61 DNC donors. After BD, SNO-Hb levels were determined to be significantly decreased compared to healthy humans (*p* = 0·003) and remained reduced for the duration of the monitoring period. There was a positive correlation between SNO-Hb and StO_2_ (*p* < 0.001). Furthermore, SNO-Hb levels correlated with and were prognostic for the number of organs transplanted (*p* < 0.001). These clinical findings provide additional support for the concept that BD induces a systemic impairment of S-nitrosylation that negatively impacts StO_2_ and reduces organ yield from DNC human donors. Exogenous S-nitrosylating agents are in various stages of clinical development. The results presented here suggest including one or more of these agents in donor support regimens could increase the number and quality of organs available for transplant.

## Introduction

Surgical and immunological techniques have advanced significantly since the first successful solid organ transplant was conducted in 1954. What has not kept pace is/are the evolution of procedures and interventions used to systemically-support the deceased by neurologic criteria (DNC) donor^1–6^. And yet it is becoming very obvious that the functional status of gifted organs at the time of procurement is/are very important to the success of both short and long-term post-graft function^7^. In this regard, we have raised the idea that disorders in organ function are a reflection of systemic physiologic failure not the result of disparate organs responding to the loss of central nervous system (CNS) regulation^8,9^. Specifically, that systemic organ failure may be due to disruptions in microvascular tissue oxygen delivery.

While DNC donor care protocols have been standardized throughout the UNOS and OPO networks, they are almost exclusively directed towards stabilizing macro-hemodynamic parameters. A consequence of this limited focus is that commonly used interventions (e.g. increasing FiO_2_; administration of vasopressors) may benefit some organs at the costs of damaging others (viz. lung and heart, respectively). No current intervention or donor support methodology specifically targets the microvascular circulation even as it is tissue oxygenation not blood oxygen saturation that impacts systemic organ function. Local tissue perfusion is regulated by a physiological response termed hypoxic vasodilation in which tissue oxygen requirements are directly coupled to blood flow, the domain of nitric oxide (NO) bioactivity^10,11^. Within red blood cells (RBCs), hemoglobin (Hb) serves as an oxygen sensor and as a hypoxia-responsive transducer of NO signals, which couples blood flow to tissue oxygen requirements^10,12,13^. In particular, vasodilation by S-nitrosylated-Hb (SNO-Hb; i.e. release of NO bioactivity) is linked to Hb desaturation and thus provides a regulated mechanism that matches blood flow and oxygen delivery to local metabolic demand^10,12^.

Decreased levels of SNO-Hb have been observed in a disparate collection of human diseases characterized by tissue hypoxemia^10^. Such findings suggest that RBC-derived NO bioactivity plays an important role in the respiratory cycle and that impairment of this activity contributes to the pathophysiology of ischemic conditions, including BD. In a translational pre-clinical study utilizing swine, we confirmed that BD resulted in reduced circulating levels of SNO-Hb, reductions in microvascular tissue oxygenation (StO_2_), and declines in systemic organ function^14^. We followed this with a study of DNC donors where we determined that StO_2_, but not arterial blood oxygenation (SaO_2_; a macrohemodynamic parameter), was directly correlated with transplantable organ yield^15^. In this current report, we link the two findings by showing SNO-Hb levels correlate directly with StO_2_ and with subsequent organ yield. The importance of this relationship lies in the fact that SNO-Hb is a druggable target and we have clinically-tested S-nitrosylating agents that can raise SNO-Hb levels, supporting the concept that such interventions could be used to increase the number and functional status of solid organs procured from DNC donors.

## Results

### Demographics

We enrolled 61 DNC donors in this study. Two subjects were excluded from the final data set due to diagnosed communicable systemic infections that prevented organ utilization and whose blood samples the research laboratory was not equipped to safely process as part of the SNO-Hb analysis. Donor demographic, physiologic data, and procurement outcomes are presented in Table 1. As a group, the subjects were well-ventilated and overall hemodynamically-stable as evidenced by constant levels of vasopressor requirement during the support phase and stable ventilator parameters and P/F ratios.

**Table 1:** Donor demographics, clinical status, procurement outcomes.

Parameter	Overall
Enrolled	61
Completed study	59
Age (median [IQR])	44 [27, 56]
Gender = female (%)	37
**Race (%)**	
Caucasian	54
African-American	36
Other	10
BMI (median [IQR])	28 [23, 32]
**Cause of death (%)**	
Anoxia	31
Trauma	44
Stroke	24
Other	1
**Donor support and outcomes**	
# of pressors (median [IQR])	2 [2]
# of transfusions (median [IQR])	0 [0, 2]
Steroids = yes (%)	81
Antibiotics = yes (%)	97
T3 = yes (%)	67
pH (median [IQR])	7.36 [7.33, 7.40]
pCO_2_ (median [IQR])	41 [37, 44]
pO_2_ (median [IQR])	145 [127, 171]
SaO_2_ (median [IQR])	98 [97, 99]
FiO_2_ (median [IQR])	0.55 [0.40, 0.70]
P/F ratio (median [IQR])	325 [217, 387]
Total support time (h) (median [IQR])	56 [36, 85]
Number of organs expected (median [IQR])	6 [4, 8]
Number of organs procured (median [IQR])	3 [3, 6]
Number of organs transplanted (median [IQR])	3 [1, 6]

### RBC SNO-Hb and tissue oxygen saturation during donor support

**Figure 1:**
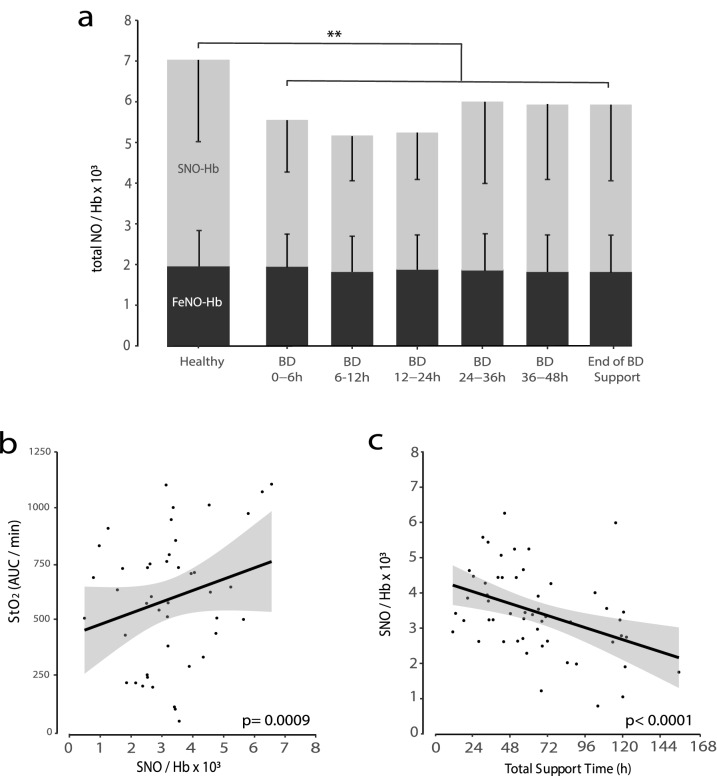
(**a**) Amount of NO bound to Hb (in form of SNO-Hb and FeNO-Hb) in DNC organ donors in 6–12 h intervals from the initiation of donor management in ICU until end of support (p > 0.1), compared to n = 10 healthy volunteers (p = 0.003). (**b**) Association between tissue oxygen saturation (StO_2_) (Area under the curve over minutes) and SNO-Hb concentration. (**c**) Association between SNO-Hb concentration and total support time (in hours).

As predicted from our pre-clinical work, we found that total RBC HbNO levels were significantly decreased in the DNC donors compared to the concentrations quantified in arterial blood aliquots obtained from a group of healthy humans (Fig. 1a;* p* = 0.003). This reduction was completely accounted for by major declines in SNO-Hb (grey columns) for the duration of the sampling period with little to no change in the circulating amounts of FeNO-Hb (black columns).

Within the DNC group, there were variations in the SNO-Hb concentrations that were directly correlated to differences in StO_2_ (Cor. Coef. 1.09, r = 0.44, Fig. 1b), i.e. lower SNO-Hb levels were associated with lower measures of tissue oxygenation consistent with the major role played by RBC-derived NO-bioactivity in regulating microvascular blood flow and oxygen delivery. Furthermore, there was a negative correlation between SNO-Hb levels and the duration of donor support (Cor. Coef. 0.98, r = − 0.39, Fig. 1c). Additionally, we did not observe any statistically significant correlation between duration of support phase and number of organs transplanted.

### Relationship between SNO-Hb and organ yield

**Figure 2:**
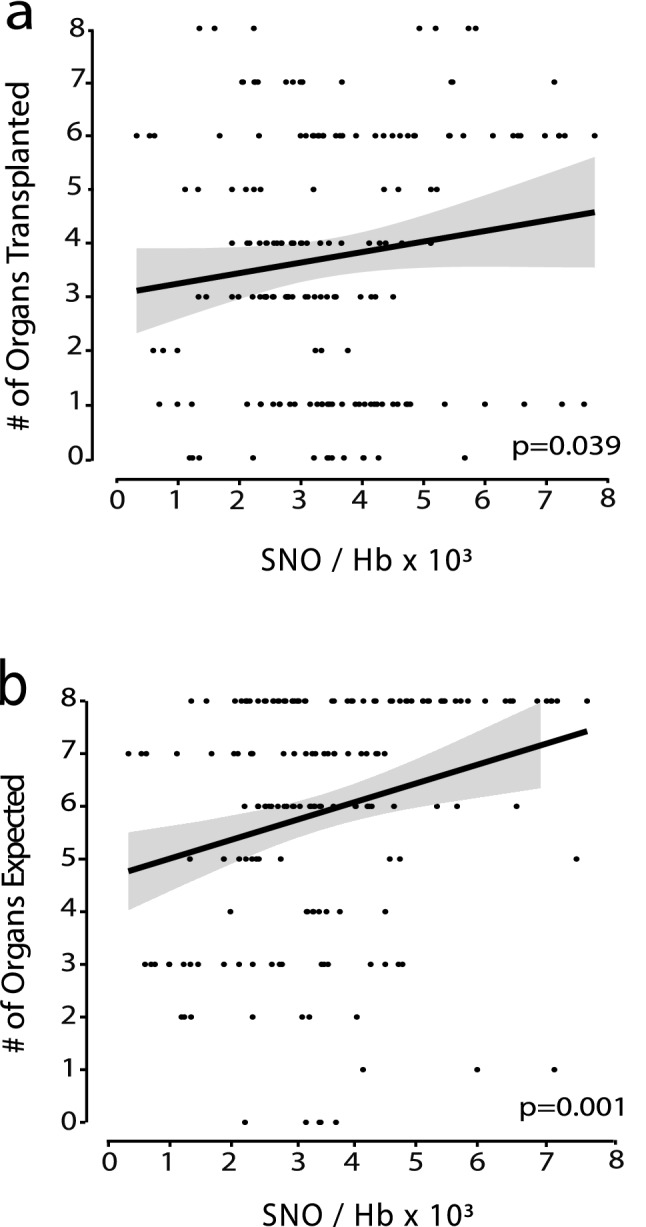
Association between amount of SNO-Hb and (**a**) number of organs transplanted, and (**b**) expected organs with Poisson regression model.

With a link previously established between StO_2_ and organ yield^15^, and the current findings of a correlation between StO_2_ and SNO-Hb levels, we next analyzed the relationship between SNO-Hb and procurement of organs deemed suitable for transplant. In univariate models, as a whole, we determined there was a direct correlation between SNO-Hb and the number of transplanted organs (Cor. Coef. 1.05, r = 0.12, Fig. 2a). This positive relationship was strengthened when the ordinate was replaced with the expected number of transplanted organs (calculated by UNOS criteria) while keeping the abscissa as donor SNO-Hb levels (Cor. Coef. 1.39, r = − 0.24, Fig. 2b). The overall relationship was further strengthened by determining that the concentrations of SNO-Hb inversely correlated with the number of organs deemed unsuitable for transplant at the end of the donor support period (Cor. Coef. 0.87, r = − 0.13, p < 0·05). In multivariate models, adjusting for SNO-Hb, age and BMI, the correlation remained statistically significant (p < 0.05). As noted in the methods, the SNO-Hb analysis and tissue oxygenation calculations were conducted off-line so neither changes in RBC-derived NO bioactivity nor StO_2_ factored into the procurement teams’ decisions regarding organ utilization or rejection.

## Discussion

Transplantation is an accepted intervention to correct innate organ failure, but current replacement needs greatly outpace organ availability^6^. Significant societal benefit would result from increasing the number of organs available for transplant. Unfortunately, donation rates for deceased donors have been static for several years despite various public education campaigns. While it is true that the proportion and number of living donors has increased, this latter group is limited to kidneys and livers and still only makes up a small percentage of the overall donor pool^16^. Further exacerbating this situation is that the number of organs recovered per donor is far less than 100%. Optimizing donor status is an obvious (if underutilized) strategy to increase the number of transplantable organs^1–3,5^ although the actual means to accomplish this goal—correcting the physiological instability produced by BD—remain unclear^1–3^. Our data support the idea of targeting the microvasculature to increase organ yield by correcting reductions in circulating NO-bioactivity. We recognize that if this was a stand-alone report, concerns regarding sample size and the correlational analyses would decrease the scientific merit of the current findings. However, we reiterate how the results presented herein complement and build upon our previous translational and clinical activities that all point to disruptions in SNO-mediated microvascular oxygen delivery as a systemic response to BD that impacts transplantable organ function during the donor support phase.

It is increasingly apparent that an extensive array of cellular activities controlled by NO are regulated by protein S-nitrosylation and that disruptions in these processes are an important contributor to a myriad of diseases and pathologic conditions, including reductions in oxygen delivery^10–14,17–23^. With specific reference to BD, the current findings, along with previously acquired data, present compelling evidence that disruptions in SNO homeostasis have significant negative impacts on DNC organ status^14,20^. Additionally, the negative correlation between SNO-Hb levels and the duration of donor support could indicate that the methodologies currently used to maintain systemic organ function do not regenerate SNO-Hb levels and may actually exacerbate the BD-induced disruptions in SNO homeostasis. Our study did not show any positive correlation between duration of support and improved organ yield both in the univariate and multivariate analyses. This is in contrast with reports that longer durations of support might improve outcome or at least showed no inferiority, especially for kidney grafts but such studies are retrospective (subject to selection bias, as the organs might have been refused for transplant, younger donors etc.) or from preclinical work.

We have actively promoted this area of S-nitrosylation research and have proposed that restoration of SNO levels may provide a novel targeted therapeutic approach to restore physiologic status in DNC donors^14,20^. As our group conducts investigations delineating pathophysiologic conditions that can be attributed (at least in part) to disruptions in NO-bioactivity and SNO-homeostasis, we have sought to identify S-nitrosylating agents suitable for clinical use^14,19,20,23^. Ethyl nitrite (ENO) is a first in class agent that readily forms SNOs, most notably SNO-Hb. In clinical studies, we have determined that inhalation of ENO to hypoxic healthy human volunteers (FiO_2_ = 0.12) increases SNO-Hb levels and improves StO_2_ in the absence of a rise in oxygen availability (i.e. SaO_2_ stayed constant). Other work has demonstrated acute oxygenation benefits in distinct clinical populations, including infants with persistent pulmonary hypertension of the newborn and adults newly diagnosed with idiopathic pulmonary arterial hypertension^10^. Of direct relevance to the current study, we recently determined in a pre-clinical swine BD preparation that inhalation of ENO improved systemic physiologic status^14^. We have also demonstrated that addition of ENO to the preservation solution of pumped human kidneys (procured but subsequently during storage were deemed unsuitable for transplant) restored cellular SNO tissue status, reduced markers of inflammation, and improved perfusion parameters to transplantable levels^20^.

The significance of these findings is two-fold:

1. disruptions in SNO homeostasis continue into the ex vivo storage/transport phase; and
2. exogenous S-nitrosylation therapy could provide functional benefits both during and after the whole-body donor support phase.

At the same time, it needs to be emphasized that predicting transplant success based upon pre-procurement SNO activity is premature. There are a variety of ex vivo storage and treatment strategies that may or may not correct SNO deficits. These include static versus active storage, hypothermic versus normothermic storage conditions, and “revitalization” interventions immediately prior to engraftment. As a result, we have deliberately limited the present results to procurement of organs deemed suitable for transplant (even as we actively explore the therapeutic potential of ex vivo interventions to improve organ status).

In conclusion, we provide clinical evidence for the concept that BD induces a systemic impairment of S-nitrosylation that negatively impacts StO_2_ and reduces organ yield from DNC human donors. Currently, S-nitrosylating agents are undergoing clinical testing to correct oxygenation pathologies^19^. This specific type of directed intervention has the potential to increase the quality and quantity of organs available for transplant and ultimately improve the outcome of transplant recipients.

## Materials and methods

### Study population

This prospective observational study replicated the design of our previous monitoring trial^15^. The target population was consented DNC organ donors ≥ 15 years of age and weighing ≥ 45 kg. Living donors and individuals declared deceased after circulatory determination of death were not studied. The study protocol was cleared by the Institutional Review Board of University Hospitals-Cleveland Medical Center, an AAHRPP-accredited institution, as non-living subjects research. In addition, the donor study was approved by the Medical Advisory Board and the Board of Directors of the local organ procurement organization for Northeast Ohio (Lifebanc; Cleveland, OH). Authorization for research participation was obtained by either pre-morbid first-person informed consent or from the donor’s next-of-kin as a component of the organ donation informed consent process. The procurement of blood samples from healthy subjects was covered under a human studies protocol approved by the UH-CMC IRB. Written informed consent was obtained from each living individual prior to their voluntary participation in the study. All experiments on living subjects were performed in accordance with relevant guidelines and regulations. Based on our previous monitoring trial^15^, we determined a sample size projection of 60 donors would yield 90% power to detect a clinically meaningful difference in organ yield. We included all eligible DNC organs from 2015 to 2018 in the study.

### Study protocol

Research personnel were contacted about eligible subjects by the Lifebanc donor management team; blood sampling and monitoring was initiated as soon as possible after obtainment of donor consent. Arterial blood sample were obtained from existing vascular monitoring lines at 6–12 h intervals. Tissue oxygenation (StO_2_) was recorded from the thenar eminence using a NIRS probe (Hutchinson Technology Inc; Hutchinson, MN). The StO_2_ values were not used to influence donor management though the care team was instructed on how to reattach the probe in case of accidental displacement. Sampling and monitoring were continued up until the point of organ retrieval. In living human volunteers, arterial blood samples were obtained by needle puncture or following placement of avascular access catheter by suitably trained physicians. In both settings, lidocaine was used to provide local pain control; no adverse events occurred. The procured blood was then processed similar to samples obtained from the DNC donor cohort.

### Clinical chemistry and other measurements

Blood gas parameters were measured using an IRMA TruPoint Blood Analysis System (LifeHealth; Roseville, MN). SNO-Hb and FeNO-Hb (defined as biologically inert heme-bound NO) levels were quantified using mercury-coupled photolysis chemiluminescence; each sample was assayed in triplicate^24,25^. Additional blood chemistries were measured by the core clinical laboratory of the supporting hospital. Donor demographics, physiologic status, organ retrieval numbers, and interventions conducted during the management phase were collected post-hoc from the subjects’ medical records. Number of organs expected were based on the OPTN-SRTR “Organ Yield Calculator”.

### Data analysis

For continuous parameters (viz. StO_2_), medians were calculated at 1-h intervals and area under the curve values were compared. Quantitative variables are presented as means ± standard deviations (SDs) for normally distributed data and median with inter-quartile ranges (IQRs) for non-normally distributed data are reported.

For comparisons, T-test and Wilcoxon–Mann–Whitney test were used for quantitative data. For qualitative variables, Chi-Square and Fisher exact tests were employed. Longitudinal analyses of repeated-measured data were done by linear mixed model with the assumption of missing data at random. For determining association between variables and the count outcome, Poisson regression modeling was implemented. For assessing linear correlations between continuous variables, the average value of each variable during recovery phase was used and Pearson method was employed. All data were analyzed with latest version of R statistical software (4·0·3) (https://www.r-project.org/). Statistically significant differences were presumed when p < 0.05.
